# Restoring the impaired cardiac calcium homeostasis and cardiac function in iron overload rats by the combined deferiprone and N-acetyl cysteine

**DOI:** 10.1038/srep44460

**Published:** 2017-03-13

**Authors:** Suwakon Wongjaikam, Sirinart Kumfu, Juthamas Khamseekaew, Siriporn C. Chattipakorn, Nipon Chattipakorn

**Affiliations:** 1 Cardiac Electrophysiology Research and Training Center, Faculty of Medicine, Chiang Mai University, Chiang Mai, Thailand; 2 Cardiac Electrophysiology Unit, Department of Physiology, Faculty of Medicine, Chiang Mai University, Chiang Mai, Thailand; 3 Center of Excellence in Cardiac Electrophysiology Research, Chiang Mai University, Chiang Mai, Thailand; 4 Department of Oral Biology and Diagnostic Sciences, Faculty of Dentistry, Chiang Mai University, Chiang Mai, Thailand

## Abstract

Intracellular calcium [Ca^2+^]_i_ dysregulation plays an important role in the pathophysiology of iron overload cardiomyopathy. Although either iron chelators or antioxidants provide cardioprotection, a comparison of the efficacy of deferoxamine (DFO), deferiprone (DFP), deferasirox (DFX), N-acetyl cysteine (NAC) or a combination of DFP plus NAC on cardiac [Ca^2+^]_i_ homeostasis in chronic iron overload has never been investigated. Male Wistar rats were fed with either a normal diet or a high iron (HFe) diet for 4 months. At 2 months, HFe rats were divided into 6 groups and treated with either a vehicle, DFO (25 mg/kg/day), DFP (75 mg/kg/day), DFX (20 mg/kg/day), NAC (100 mg/kg/day), or combined DFP plus NAC. At 4 months, the number of cardiac T-type calcium channels was increased, whereas cardiac sarcoplasmic-endoplasmic reticulum Ca^2+^ ATPase (SERCA) was decreased, leading to cardiac iron overload and impaired cardiac [Ca^2+^]i homeostasis. All pharmacological interventions restored SERCA levels. Although DFO, DFP, DFX or NAC alone shared similar efficacy in improving cardiac [Ca^2+^]i homeostasis, only DFP + NAC restored cardiac [Ca^2+^]i homeostasis, leading to restoring left ventricular function in the HFe-fed rats. Thus, the combined DFP + NAC was more effective than any monotherapy in restoring cardiac [Ca^2+^]_i_ homeostasis, leading to restored myocardial contractility in iron-overloaded rats.

Iron overload cardiomyopathy is the primary cause of death in hereditary hemochromatosis^1^,^2^ and transfusion dependent thalassemia (TDT) patients^3^,^4^. Non-transferrin bound iron (NTBI) occurs when transferrin becomes saturated and can therefore no longer bind free iron^5^,^6^. NTBI is represented in the plasma of iron-overloaded patients when transferrin saturation is more than 70% and which two conditions are associated with iron overload cardiomyopathy in thalassemic patients^7^,^8^. In addition, labile plasma iron (LPI) is a redox active, and a labile-chelatable component of NTBI, which is presented in the plasma of TDT and non-transfusion dependent thalassemia (NTDT) patients^7^,^9^,^10^,^11^. Excess plasma NTBI and LPI leads to iron overload, both systemically and within tissues, resulting in increased reactive oxygen species (ROS) via Haber-Weiss and Fenton’s reactions^6^,^7^. Increased ROS is a major cause of tissue and organ damage, particularly in the heart^6^,^12^. Plasma NTBI and LPI can rapidly enter cardiomyocytes, resulting in increased free iron or labile cellular iron, which causes increased cardiac oxidative stress and iron overload cardiomyopathy^7^. L-type calcium (Ca^2+^) channels (LTCC) and T-type Ca^2+^ channels (TTCC) are voltage-gated ion channels which are abundantly expressed in the heart, and play an important role in the excitation–contraction coupling of cardiomyocytes^13^,^14^. Previous studies found that LTCC and TTCC are major portals for iron entry into the heart in iron overload cardiomyopathy^15^,^16^,^17^,^18^,^19^. In addition, TTCC exist and contribute to cardiac electrical activity during the early embryonic state^20^ and disappear shortly after birth so they cannot be found in adult ventricular cardiomyocytes under physiological conditions^21^,^22^. However, TTCC reappear in ventricular myocytes under some pathological conditions such as ventricular hypertrophy^23^, post myocardial infarction^22^, and iron overload^18^,^19^. Previous studies have demonstrated that an increase in cardiac iron concentration depended on Fe^2+^ influx through either LTCC or TTCC, leading to abnormal cardiac contractility and cardiac dysfunction under conditions of iron overload^15^,^16^,^18^,^19^. Increased ROS production mediated by iron overload can directly affect key regulators of excitation-contraction coupling, in particular sarcoplasmic-endoplasmic reticulum Ca^2+^ ATPase (SERCA) and/or sodium- Ca^2+^ exchangers (NCXs), which leads to impaired cardiac intracellular Ca^2+^ [Ca^2+^]i transients causing diminished cardiac contractility and heart failure^2^,^24^. Additionally, SERCA is very sensitive to oxidative stress leading to protein damage and dysfunction which can be a major cause of increased diastolic Ca^2+^ level and prolonged Ca^2+^ decay rate leading to cardiac dysfunction and heart failure under the conditions of iron overload^2^,^25^.

There are several iron regulatory proteins which monitor iron metabolism in the heart. Divalent metal transporter 1 (DMT1) has an important role in the uptake of iron into the heart^26^. However, studies on the function of DMT1 in the iron-overloaded heart have inconsistent findings. A study found that iron overload could suppress the level of cardiac DMT1 protein but it had no significant effect on DMT1 mRNA expression in the heart of the rats^27^. On the contrary, a few studies found that the level of DMT1 protein did not alter in the hearts of rats^26^ and thalassemic mice^17^ under conditions of iron overload. Zrt- and Irt-like protein 14 (Zip14) are abundantly expressed in the liver, pancreas and heart^26^,^28^. A previous study found that Zip14 mediated the uptake of zinc and NTBI into the liver^29^ and it may also mediate the uptake of NTBI into the heart. In addition, hepcidin and ferroportin are iron regulatory proteins which play an important role in iron homeostasis. Previous studies demonstrated that hepcidin and ferroportin are presented in the heart^17^,^30^. Ferroportin functions to export iron when iron is too abundant in the heart^17^,^30^. On the other hand, hepcidin is a negative regulator of iron metabolism by promoting ferroportin internalization and degradation^31^,^32^ leading to reduced cardiomyocyte iron efflux^30^. Additionally, hepcidin functions in a decreased dietary iron absorption form duodenal enterocyte under iron overload conditions leading to iron homeostasis^33^,^34^.

At the present time, three frequently used iron chelators; subcutaneous deferoxamine (DFO), oral deferiprone (DFP) and oral deferasirox (DFX), are used to treat patients with iron overload cardiomyopathy^35^,^36^,^37^. Nevertheless, a head to head comparison of the comparative therapeutic effects between DFO, DFP or DFX on cardiac [Ca^2+^]i homeostasis as well as calcium cycling and iron regulatory proteins in iron-overloaded rats has never been investigated. In addition, growing evidence indicates that N-acetyl cysteine (NAC) has an iron chelating property^38^, and is a potent antioxidant which is used to treat children with β-thalassemia^39^. Moreover, our recent study also found that combined DFP plus NAC had synergistic beneficial effects in restoring function to the brain^40^,^41^ and heart^42^ in iron-overloaded rats. However, the effects of combining an oral iron chelator DFP and an antioxidant NAC on cardiac [Ca^2+^]i homeostasis as well as the impact of this treatment on calcium cycling and iron regulatory proteins in iron-overloaded rats have never been investigated. The aim of this study was to investigate the hypothesis that DFO, DFP, DFX or NAC alone can improve cardiac [Ca^2+^]i homeostasis and left ventricular (LV) function in iron-overloaded rats, as well as whether the combined DFP plus NAC treatment can synergistically provide beneficial effects for these conditions.

## Results

### Effects of the pharmacological interventions on plasma NTBI and MDA levels, and cardiac iron and MDA concentrations

Plasma NTBI level was significantly increased in the HFeV rats when compared with the NDV rats, indicating that an iron overload condition occurred in the HFe-fed rats (Supplementary Table 1). On the other hand, plasma NTBI level was significantly decreased in iron-overloaded rats treated with DFO, DFP, DFX, NAC and combined DFP plus NAC treatment for 2 months (Supplementary Table 1). In consistency with plasma NTBI level, plasma MDA level was significantly increased in the HFeV rats, when compared with the NDV rats, indicating that plasma oxidative stress occurred in the HFe-fed rats (Supplementary Table 1). All pharmacological interventions significantly decreased plasma MDA level in iron-overloaded rats after 2 months of treatment. In addition, the cardiac iron status showed that cardiac iron concentration was significantly increased in the HFeV rats when compared with the NDV rats (Supplementary Table 1). DFO, DFP or DFX alone decreased cardiac iron concentration, and NAC exerted iron chelating properties as it also lowered cardiac iron concentration in iron-overloaded rats (Supplementary Table 1). However, only combined DFP plus NAC significantly decreased cardiac iron concentration to a normal level as shown in the NDV rats (Supplementary Table 1). Also, cardiac MDA concentration was significantly increased in the HFeV rats when compared with the NDV rats (Supplementary Table 1). Although monotherapy alone could reduce cardiac MDA concentration, combined DFP plus NAC reduced cardiac MDA concentration to a normal level as shown in the NDV rats (Supplementary Table 1). These results suggest that a combination therapy of DFP and NAC can effectively restore cardiac iron concentration and oxidative stress in iron-overloaded rats.

### Effects of the pharmacological interventions on cardiac function

**Figure 1:**
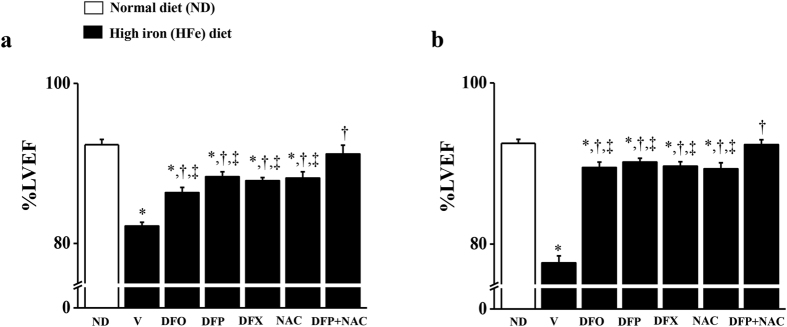
Combined DFP + NAC restores left ventricular (LV) function in iron-overloaded rats. The effects of the pharmacological interventions on percentage of LV ejection fraction (%LVEF) at 3 months (**a**) and 4 months (**b**), respectively in iron-overloaded rats. *P < 0.05 vs. ND control, ^†^*P* < 0.05 vs. HFeV, ^‡^*P* < 0.05 vs. HFeDFP + NAC.

Chronic iron overload led to cardiac dysfunction in iron-overloaded rats. The percentage of LV ejection fraction (%LVEF) decreased markedly in the HFeV rats at 3 months (Fig. 1a), and 4 months (Fig. 1b) when compared with the NDV rats at 3 and 4 months, respectively. At 3 months (after 1 month of treatment with all pharmacological interventions), DFO, DFP, DFX or NAC monotherapy significantly increased %LVEF in the HFe-fed rats, while only the combination therapy of DFP and NAC led to significant increases in %LVEF, attaining a normal level similar to the NDV rats (Fig. 1a). Consistently, %LVEF was similar after 2 months of treatment (at the end of the experiment) with all pharmacological interventions in iron-overloaded rats (Fig. 1b). The results suggest that combined DFP plus NAC can effectively restore cardiac function in iron-overloaded rats which exerts greater cardioprotective effects than either DFO, DFP, DFX or NAC monotherapy after 1 month of treatment.

### Effects of the pharmacological interventions on cardiac [Ca^2+^]i homeostasis

**Figure 2:**
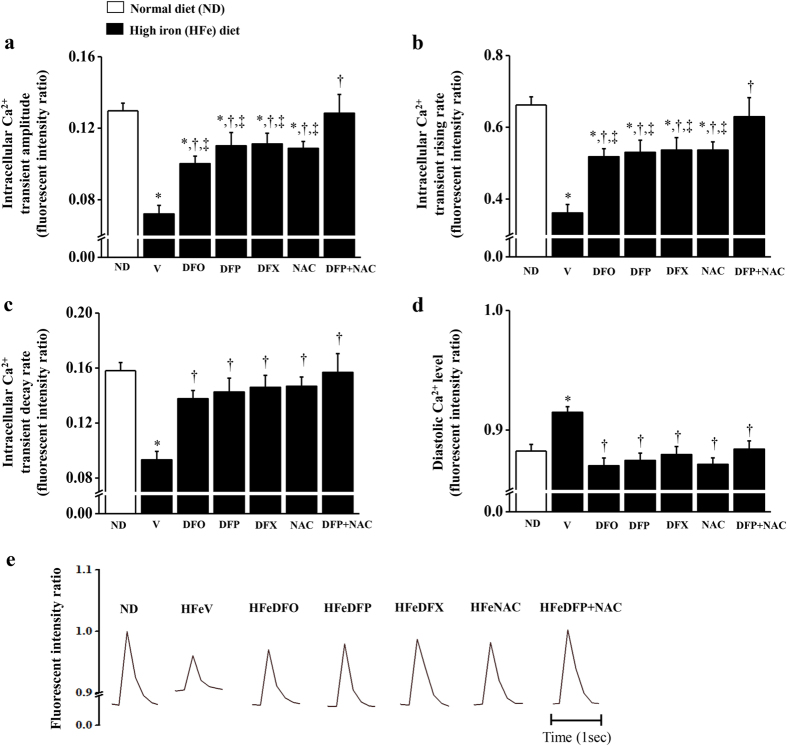
Combined DFP + NAC restores calcium homeostasis in iron-overloaded rats. The effects of the pharmacological interventions on cardiac intracellular calcium transients: (**a**) intracellular Ca^2+^ transient amplitude; (**b**) intracellular Ca^2+^ transient rising rate; (**c**) intracellular Ca^2+^ transient decay rate; (**d**) diastolic Ca^2+^ level, and (**e**) calcium tracing in iron-overloaded rats. *P < 0.05 vs. ND control, ^†^*P* < 0.05 vs. HFeV, ^‡^*P* < 0.05 vs. HFeDFP + NAC.

An impaired level of cardiac [Ca^2+^]i transients occurred in iron-overloaded rats when compared with the NDV rats (Fig. 2a–e). The results found that cardiac [Ca^2+^]i transient amplitude, rising rate and decay rate were all significantly decreased, whilst diastolic Ca^2+^ levels were significantly increased in the HFeV rats, indicating cardiac [Ca^2+^]i dyshomeostasis, when compared with the NDV rats (Fig. 2a–e). After 2 months of treatment, DFO, DFP, DFX or NAC alone caused improved [Ca^2+^]i transient amplitude and rising rate, while only combined DFP plus NAC led to restored normal [Ca^2+^]i transient amplitude and rising rate in iron-overloaded rats (Fig. 2a,b). On the other hand, all of the pharmacological intervention treatments led to restored [Ca^2+^]i transient decay rates and diastolic Ca^2+^ levels in iron-overloaded rats to normal levels similar to those in the NDV rats (Fig. 2c,d). The results suggest that only combined DFP plus NAC effectively restores all parameters of Ca^2+^ homeostasis in iron-overloaded rats.

### Effects of the pharmacological interventions on cardiac Ca^2+^ cycling protein

**Figure 3:**
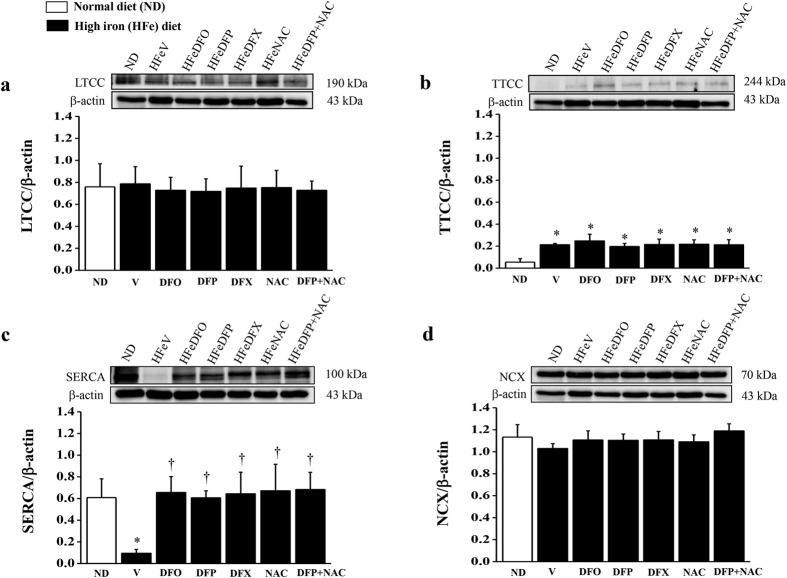
Combined DFP + NAC restores the levels of SERCA protein in iron-overloaded rats. The effects of the pharmacological interventions on the levels of calcium cycling proteins: (**a**) L-type Ca^2+^ channels (LTCC); (**b**) T-type Ca^2+^ channels (TTCC); (**c**) sarcoplasmic-endoplasmic reticulum Ca^2+^ ATPase (SERCA), and (**d**) sodium- Ca^2+^ exchangers (NCX) in iron-overloaded rats. *P < 0.05 vs. ND control, ^†^*P* < 0.05 vs. HFeV. The full-length blots are presented in Supplementary Figures 1, 2 and 3.

The levels of TTCC were significantly increased and the level of SERCA was significantly decreased in the HFeV rats when compared with the NDV rats, while the levels of LTCC and NCX did not differ between the NDV rats and iron loading groups (Fig. 3a–d). After 2 months of treatment, all pharmacological interventions led to the restoration of SERCA protein expression to a normal level in the HFe-fed rats. On the other hand, the levels of LTCC, TTCC or NCX did not alter in the HFe-fed rats after treatment with either DFO, DFP, DFX, NAC or combined DFP plus NAC for 2 months (Fig. 3a,b,d).

### Effects of the pharmacological interventions on cardiac iron transporter and regulatory proteins

**Figure 4:**
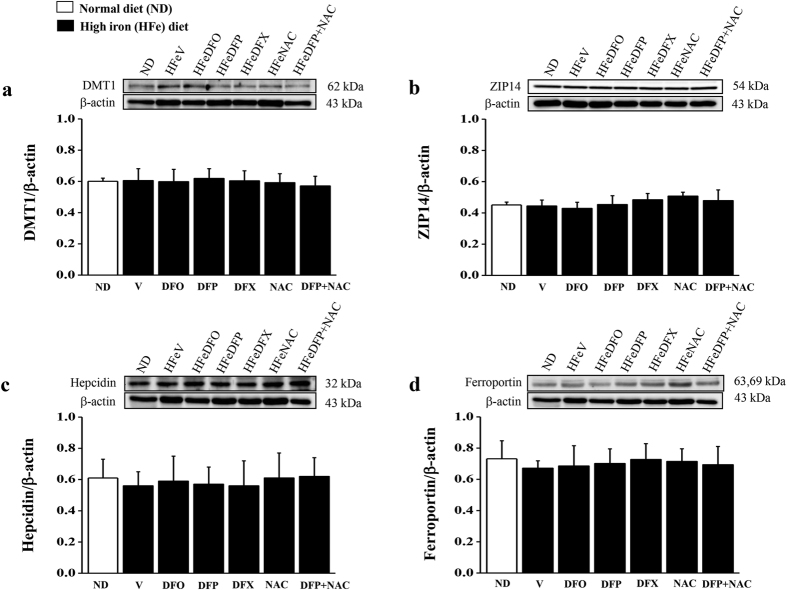
All treatments has no effect on the levels of iron transporter and regulatory proteins. The effects of the pharmacological interventions on the levels of iron transporter protein: (**a**) divalent metal transporter 1 (DMT1); (**b**) ZRT/IRT-like protein 14 (ZIP14); the levels of iron regulatory protein (**c**) hepcidin, and (**d**) ferroportin in iron-overloaded rats. The full-length blots are presented in Supplementary Figures 1, 2 and 3.

In iron-overloaded heart, the levels of cardiac iron transporter protein, including DMT1 and Zip14 as well as the levels of cardiac iron regulatory protein including ferroportin and hepcidin, did not differ in all groups of iron-overloaded rats when compared with the NDV rats (Fig. 4a–d). Similarly, all protein levels did not alter in the HFe-fed rats after 2 months of treatment with either DFO, DFP, DFX, NAC or combined DFP plus NAC when compared with the HFeV rats (Fig. 4a–d).

## Discussion

The main conclusions which could be drawn from this study were as following: (1) chronic iron overload led to increased levels of TTCC, decreased level of SERCA, and impaired cardiac [Ca^2+^]i homeostasis, including decreased [Ca^2+^]i transient amplitude, rising rate, and decay rate as well as increased diastolic Ca^2+^ levels leading to LV dysfunction; (2) DFO, DFP, DFX or NAC monotherapy could lead to the restoration of the level of SERCA protein, improvement in cardiac [Ca^2+^]i transient amplitude, rising rate as well as restoring [Ca^2+^]i transient decay rate and diastolic Ca^2+^ level leading to improved LV function in iron-overloaded rats; (3) only a combination of DFP plus NAC resulted in a restoration of all parameters of Ca^2+^ homeostasis leading to restored normal LV function in iron-overloaded rats; (4) chronic iron overload and all pharmacological interventions had no effect on the levels of DMT1, ZIP14, hepcidin, ferroportin, LTCC and NCX in iron-overloaded rats. The summary of major findings of this study is shown in Table 1.

This is the first study to demonstrate the cardioprotective effects of DFO, DFP, DFX, NAC or combined DFP plus NAC treatment on iron overload-induced cardiac [Ca^2+^]i dysregulation in rats. It is also significant that our results show that combined DFP plus NAC exerted synergistically beneficial effects on cardiac [Ca^2+^]i transients, and the combination therapy was more effective than monotherapy in iron-overloaded rats. Ca^2+^ function as secondary messengers and play an important role in many signaling pathways in all cell types^43^,^44^,^45^. In cardiomyocytes, [Ca^2+^]i regulate electrical signals which play a role in cardiac rhythm and excitation–contraction coupling leading to cardiac contraction and relaxation^43^. During cardiac action potentials, LTCCs are activated and then an influx of Ca^2+^ triggers Ca^2+^ release from the sarcoplasmic reticulum through ryanodine receptor2 (RyR2), which is called Ca^2+^ -induced Ca^2+^ release leading to directly activated cardiomyocyte contraction^43^,^46^. During cardiac relaxation, Ca^2+^ is removed from the cytosol by four types of Ca^2+^ transporters: (1) Ca^2+^ is taken up into the sarcoplasmic reticulum by SERCA; (2) Ca^2+^ is removed from cytosol by NCX, and (3) sarcolemmal Ca^2+^-ATPase; (4) Ca^2+^ is taken up into mitochondria by mitochondrial Ca^2+^ uniporters^46^. Under conditions of iron overload, LTCC and TTCC are the major pathways for iron entry into the heart leading to disturbance in cardiac contractility and iron overload cardiomyopathy^15^,^16^,^18^,^19^. Previous studies found that at a high concentration of ferrous iron (Fe^2+^), Ca^2+^ current could decrease, indicating that Fe^2+^ might compete with Ca^2+^ influx into the heart through LTCC which may contribute to systolic dysfunction in cases of iron overload cardiomyopathy^47^,^48^. Additionally, we found that the decay of the Ca^2+^ current was slower when Fe^2+^ concentration was increased in isolated ventricular cardiomyocytes of rats, which might possibly contribute to impaired diastolic function^47^,^48^. However, the mechanism of extracellular Fe^2+^ entry into the cardiomyocytes through TTCC in iron overload conditions may be similar to LTCC. As expected, a previous study found that Ca_V_3.1 TTCC was a significant portal for Fe^2+^ entry into cells leading to an iron overload condition^49^. Consistent with a recent study indicated that at a high concentration of free Fe^2+^ may compete with a Ca^2+^ influx into the myocardium through Ca^2+^ channels by both LTCC and TTCC^50^. Our previous study also found that TTCC is an important pathway for Fe^2+^ entry into the heart of thalassemic mice under iron overload conditions^17^,^18^,^19^. Hence, elevated TTCC expression in this study indicates that it may be an important portal for iron uptake into the cardiomyocytes and causes an increased cardiac iron concentration in rats suffering from iron-overload. Although iron overload did not alter the level of cardiac LTCC protein in this study, LTCC activity might be altered in the hearts of the HFe-fed rats and this needs to be investigated further. In addition, our previous study found that rats with iron-overload for 4 months had both increased systemic iron (plasma NTBI) and cardiac iron concentrations^42^ which were consistent with an increase in TTCC protein expression in this study. Plasma NTBI levels were used to determine an iron status in iron-overloaded patients including hereditary hemochromatosis, β-thalassemia, myelodysplastic syndrome and sickle cell disease^51^. However, the mere presence of NTBI in plasma of patients with iron overload might not be sufficient for causing heart iron overload as found in hypertransfused sickle cell anemia^52^, and in most cases of myelodysplastic syndrome^53^. A previous study found that heart disease was not present in patients with thalassemia major and intermedia who showed negative plasma NTBI and had a plasma transferrin saturation of less than 70%^8^. On the other hand, all thalassemic patients with heart disease found that they were plasma NTBI positive and had a plasma transferrin saturation above 70%^8^. However, several previous studies noted that LPI is found in iron-overloaded patients, which is used as a marker of iron toxicity^7^,^9^,^10^,^11^. An increase in LPI leads to increased labile cellular iron in the heart, resulting in increased cardiac oxidative stress and cardiomyopathy^7^. In addition, glutathione (GSH) system is an endogenous antioxidant that plays an important role in scavenging cellular oxidative stress, particularly in an iron overload condition^54^. Oxidative stress can occur in systemic iron overload, caused by a decrease in the levels of antioxidants, such as GSH and glutathione peroxidase (GPX), and an increase in highly toxic free radicals such as hydroxyl radical and superoxide anion^7^,^54^. Therefore, LPI and those of GSH levels should be further determined to warrant the levels of free iron and antioxidant as well as oxidative stress in plasma and heart tissues in our iron-overloaded animals.

Iron-catalyzed Haber-Weiss and Fenton’s reactions led to increased ROS production in iron overloaded rat’s plasma and heart tissue^42^. Iron overloading mediated oxidative stress and altered cardiac excitation-contraction coupling which contributed to decreased systolic and increased diastolic Ca^2+^ levels, which resulted in inhibited systolic and diastolic functions; a characteristic of iron overload cardiomyopathy^2^,^48^. Defects of calcium cycling protein including LTCC, RyR2, SERCA2a and NCX were the result of impaired calcium signaling and led to heart failure^43^. Importantly, a previous study indicated that chronic iron overload led to reduced levels of SERCA2a in both its mRNA and protein form, resulting in abnormal Ca^2+^ cycling and decreased cardiac function^2^. Consistent with this line of reasoning the level of cardiac SERCA protein was significantly decreased in iron-overloaded rats which could have led to the resulting impaired cardiac [Ca^2+^]i transients and cardiac contractility in the present study. Impaired cardiac SERCA function could prolong [Ca^2+^]i transient decay rates, leading to reduced Ca^2+^ reuptake into the sarcoplasmic reticulum and resulting in increased diastolic Ca^2+^ level in the present study. In addition, a previous study found that Fe^2+^ could directly inhibit the binding of [3H] ryanodine to its high affinity sites on RyR2 which led to reduced sensitivity of cardiac ryanodine receptors towards activation by Ca^2+^ 55. This same study indicated that Fe^2+^ competed with Ca^2+^ at the activating sites on RyR2 leading to decreased Ca^2+^-induced Ca^2+^ release in rat hearts which might contribute to impaired cardiac [Ca^2+^]i homeostasis, and cardiac dysfunction under conditions of iron overload^55^. Therefore, iron overload might subsequently result in reduced Ca^2+^ release by the sarcoplasmic reticulum leading to decreased [Ca^2+^]i transient amplitude and rising rate, and caused impaired cardiac contractility in the present study. Finally, %LVEF was significantly decreased in the HFe-fed rats leading to LV dysfunction in our study. In addition, our previous study and others demonstrated that an increase in cardiac iron store led to increased cardiac oxidative stress which caused increased mitochondrial dysfunction, resulting in cardiac dysfunction and iron overload cardiomyopathy^7^,^42^. In consistency, a previous study found that iron overload-evoked production of cardiac oxidative stress contributed to damage to mitochondrial DNA, reduced complex I (NADH dehydrogenase) and complex IV (cytochrome c oxidase) activities, and decreased cardiac mitochondrial respiration, leading to mitochondrial and cardiac dysfunctions in iron-overloaded mice^56^. Hence, cardiac mitochondrial dysfunction evoked by chronic iron overload might cause reduced ATP production in cardiomyocytes^7^. In addition, SERCA uses the free energy of ATP to transport Ca^2+^ into the sarcoplasmic reticulum through a concentration gradient which leads to intracellular Ca^2+^ homeostasis^57^. This may lead to a decrease in ATP levels in cardiac iron overload and cause reduced SERCA function and/or activity, which results in impaired Ca^2+^ homeostasis as shown in our present study. Therefore, the restoration of Ca^2+^ homeostasis in iron-overloaded heart could be an indicator of functional restoration, which might have resulted from improved ATP production and respiration, due to both iron detoxification and an improved reductive capacity by either iron chelators or antioxidant(s). Nevertheless, the effects of iron chelators and/or antioxidant(s) on cardiac mitochondrial ATP production and respiration in iron-overloaded rats should be further investigated.

However, in our present study the levels of DMT1 and ZIP14 did not alter in the HFe-fed rats when compared with the NDV rats. The results support the findings in previous studies which demonstrated that DMT1 and ZIP14 were not the common pathways for iron entry into the heart under iron overload conditions^18^,^26^. In addition, the levels of cardiac ferroportin and hepcidin also did not alter in the HFe-fed rats when compared with the NDV rats. This result was consistent with our previous study in which cardiac ferroportin and hepcidin did not alter in the wild type mice after 4 months of iron loading when compared with the ND control groups^17^. However, previous studies demonstrated that hepcidin is primarily produced and secreted by the liver leading to the regulation of iron exporter ferroportin, all of which play a major role in controlling systemic iron concentrations in conditions of iron overload^58^,^59^. Hence, ferroportin and hepcidin may not play an important role in the heart in the iron overload conditions induced in this present study.

**Figure 5:**
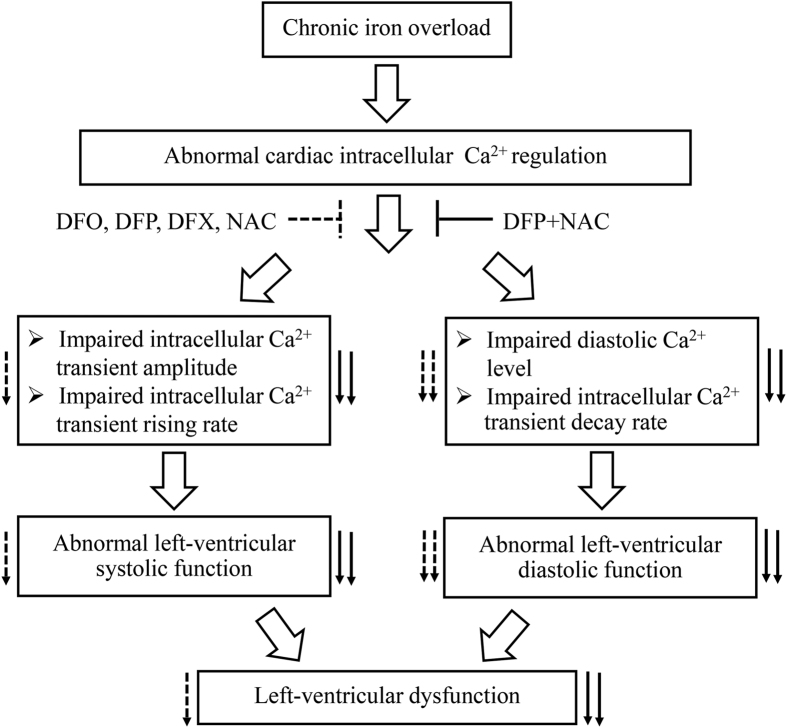
The effects of the pharmacological interventions on calcium homeostasis in iron-overloaded rats. Diagram demonstrating the suggested mechanisms of chronic iron overload on cardiac intracellular Ca^2+^ homeostasis, and left ventricular function as well as the effects of the pharmacological interventions on cardiac intracellular Ca^2+^ homeostasis, and left ventricular dysfunction mediated by chronic iron overload. Dashed arrows indicate the effects of either DFO, DFP, DFX or NAC treatment; solid arrows indicate the effects of combined DFP plus NAC treatment. Ca^2+^ = calcium; DFO = deferoxamine; DFP = deferiprone; DFX = deferasirox; NAC = N-acetyl cysteine.

Interestingly, our recent studies found that the combination of an oral iron chelator DFP and an antioxidant NAC could improve and restore normal brain and heart function effectively and provided more robust results than either DFO, DFP, DFX or NAC alone in the iron-overloaded rat model^40^,^42^. Consistently in the present study, a combination of DFP plus NAC provided more effective results than either DFO, DFP, DFX, or NAC alone in restoring cardiac [Ca^2+^]i homeostasis leading to restored normal cardiac contractility and LV function by increased %LVEF to a normal condition in iron-overloaded rats. Although previous studies reported that neither DFO nor DFX have shown to be efficient in improving %LVEF in thalassemia major or TDT patients^60^,^61^, DFP has effectively improved % LVEF in these patients^60^. On the other hand, a recent study found that although % LVEF was not altered, cardiac iron concentration was continuously reduced, and end-diastolic and end-systolic left ventricular volume were significantly diminished, leading to improved cardiac function in TDT patients after 5 years of treatment with DFX^62^. In an animal study, it has been shown that %LVEF was improved by chronic treatment with DFO, leading to improve cardiac function in iron-overloaded gerbils^63^. In consistency, our present study found that long-term treatment with DFO or DFX for 2 months could ameliorate cardiac function by increasing %LVEF, as well as improve hemodynamic parameters, including left ventricular end-systolic pressure, maximum pressure, cardiac output and stroke volume, contributing to improved cardiac function in iron-overloaded rats as shown in our previous study^42^. However, the dose and duration of treatment with DFX or DFO in TDT patients and iron-overloaded gerbils were different from our present study. Consequently, the present study indicate that chronic and continuous treatments with DFO or DFX might help to ameliorate cardiac function in patients with iron overload cardiomyopathy. The mechanisms regarding the combination of DFP and NAC in restoring cardiac [Ca^2+^]i transients could be clarified by the following reasons. First, DFP has the lowest molecule weight of iron chelators when compared with DFO and DFX, and has a high lipophilicity which means it can rapidly penetrate into cells^64^ leading to DFP chelating intracellularly effectively free irons from heart tissue^42^, and may contribute to attenuating impaired cardiac [Ca^2+^]i homeostasis. Second, NAC is an antioxidant that can decrease ROS levels, increase SERCA2a activity as well as enhance cytosolic Ca^2+^ removal^65^. In addition, NAC provides an iron chelating activity^38^ as it can remove excessive free irons out of the heart^42^ which may lead to a decrease in iron-mediated cardiac [Ca^2+^]i dysregulation in this study. Moreover, NAC has been considered and used as a promoter of GSH synthesis and thereby cell reductive power^66^. As such, it could have facilitated iron removal from iron-overloaded cells or tissues by chelators, by rendering the accumulated iron more accessible (indirectly via its reduction). Therefore, these two conditions have shown the synergistic effects of combined DFP plus NAC on cardiac [Ca^2+^]i homeostasis in the present study. The effects of all pharmacological interventions on cardiac [Ca^2+^]i homeostasis and LV function in iron-overloaded rats are summarized in Fig. 5. However, the usefulness of combined DFP plus NAC needs clinical trials to warrant its cardioprotective effects in human patients with iron overload cardiomyopathy. In conclusion, all pharmacological interventions had no effect on the levels of DMT1, ZIP14, hepcidin, ferroportin, LTCC, TTCC or NCX in the heart. However, only cardiac SERCA levels were restored by all pharmacological intervention treatments in rats with iron-overload. In addition, DFO, DFP, DFX or NAC alone exerted similar effects in improving [Ca^2+^]i homeostasis leading to improved LV function. Although these monotherapy treatments showed similar efficacy to combined DFP plus NAC in restoring [Ca^2+^]i transient decay rate and diastolic Ca^2+^ level, only combined DFP plus NAC could restore all parameters of [Ca^2+^]i transients, including [Ca^2+^]i transient amplitude, rising rate, decay rate as well as diastolic Ca^2+^ levels. These factors led to a restored normal LV function in rats with iron-overload. For this reason, combined DFP plus NAC therapy may provide these similar cardioprotective results in patients with iron overload and so may be used as a future therapeutic strategy for treatment of iron overload cardiomyopathy particularly in hemochromatosis and TDT patients.

## Methods

### Animal preparation

The Institutional Animal Care and Use Committee (IACUC) at the Faculty of Medicine, Chiang Mai University authorized the animal study protocols (Permit number: 21/2557), in compliance with NIH guidelines, and in accordance with the ARRIVE guidelines for reporting experiments involving animals^67^. The National Laboratory Animal Center, Mahidol University, Bangkok, Thailand supplied all adult male Wistar rats which had a body weight of 180–200 g An animal holding room was used to house all rats in a controlled environment of 23 ± 2 °C, 50 ± 10% humidity and 12 h light/dark cycles. Throughout the entire experiment all rats were given drinking water *ad libitum* and were acclimatized for 7 days prior to the experimental protocols.

### Experimental protocols

A standard laboratory rat diet containing 0.2% of ferrocene (C_10_H_10_Fe; Sigma-Aldrich, Co., St. Louis, USA) (w/w) was prepared as previously outlined^42^. Adult male Wistar rats were divided into 2 groups and were given either a normal diet (a chow diet, ND) (n = 12), or a high iron diet (0.2% ferrocene w/w, HFe) (n = 72) for 4 months. At 2 months, ND-fed rats had a vehicle (normal saline solution, NSS) (NDV or ND control group) administered once a day via either subcutaneous injection (n = 6) or gavage feeding (n = 6) and a normal chow diet was maintained for 2 months. The HFe-fed rats were divided into 6 groups (n = 12/group) and were given: (1) NSS (HFeV) once a day via either subcutaneous injection (n = 6) or gavage feeding (n = 6), (2) deferoxamine (DFO; Desferal^®^, Novartis Pharma Stein AG, Stein, Switzerland), (HFeDFO) 25 mg/kg/ day via subcutaneous injection, (3) deferiprone (DFP; Ferriprox^®^, Apotex Inc., Toronto, Ontario, Canada), (HFeDFP) 75 mg/kg/day, (4) deferasirox (DFX; Exjade^®^, ICL670, Novartis Pharma Stein AG, Stein, Switzerland), (HFeDFX) 20 mg/kg/day, (5) N-acetyl cysteine (NAC, Sigma-Aldrich, Co., St. Louis, USA), (HFeNAC) 100 mg/kg/day, or (6) combined DFP 75 mg/kg/day plus NAC 100 mg/kg/day (HFeDFP + NAC) through gavage feeding for 2 months, and all iron loading groups were regularly fed with the high iron diet. Since DFO is a parenteral iron chelator, it may cause suffering in patients with cardiac iron overload who are continuously treated with this drug. Previous studies have shown that DFP was significantly more potent than DFO in diminishing cardiac iron overload in TDT patients^35^,^60^. Although DFP and DFX are oral iron chelators, the cost of DFX is more expensive than DFP. Additionally, DFP was found to exert greater efficacy than DFX in reducing severe cardiac iron and improving cardiac function in TDT patients^35^,^68^. DFX also failed to reduce cardiac iron concentration in TDT patients with severe hepatic iron loading^69^. Hence, DFP was chosen in combination with NAC in this study for determining its effects on chronic iron-mediated the impairment of cardiac [Ca^2+^]i homeostasis. Dosage of all iron chelators are the lowest therapeutic range that can be used to treat patients with iron overload^36^. At the end of 3 and 4 months of the experiment, all rat groups had their cardiac function determined by echocardiography. After 4 months of the experimental period, half of the rats in each group (n = 6/group) were deeply anesthetized by intraperitoneal injections of Zolitil (ZolazepamTiletamine) 50 mg/kg combined with Xylazine 3 mg/kg^70^, then the hearts were rapidly removed. The heart tissues were frozen in liquid nitrogen immediately, and stored at −80 °C in preparation for western blot analysis. In addition, the other half of the rats from each group (n = 6/group) were deeply anesthetized with thiopental (0.5 mg/kg; Research institute of antibiotics and biotransformations, Roztoky, Czech Republic), and then the hearts of the rats were rapidly removed for single ventricular myocyte isolation^71^. Cardiomyocyte cells were isolated by using an enzymatic technique^71^,^72^. In brief, rats were injected intraperitoneally with 0.2 mL heparin for 10 minutes. After the initiation of deep anesthesia using 100 mg/kg thiopental, the heart was excised and the aorta was cannulated rapidly to enable cardiomyocyte isolation. Then, isolated cardiomyocytes from the hearts of the rats in each group (n = 5–8 cells/rat) were used for the measurement of [Ca^2+^]i transients.

### Echocardiographic studies

Cardiac function was determined using echocardiography (GE Vivid I)^42^. Echocardiographic study was performed using a method described previously^42^. The parameters from the echocardiography including LV end-systolic volume (LVESV), and end-diastolic volume (LVEDV) were determined. The percentage of ejection fraction was calculated by using the following formula: %EF (Teich) = (LVEDV-LVESV) × 100/ LVEDV^42^,^73^.

### Experimental protocol for studying cardiac [Ca^2+^]i transients

The Ca^2+^ transient level in rat ventricular myocytes was measured using a fluorimetric ratio technique^71^,^72^. The fluorescent Ca^2+^ indicator Fura-2 was loaded by incubating the cardiomyocytes at room temperature for 30 minutes with 25 μM of Fura-2-AM (Sigma Chemical, St. Louis, MO, USA). Ultraviolet light wavelengths of 340 and 387 nm were used for the excitation of the Fura-2 from a xenon arc lamp controlled by a microfluorometry system (Cell^R^, MT 20, Olympus Soft Imaging Solutions GmbH, Germany) and the excitation light beam was directed into an inverted microscope (IX-81, Olympus Tokyo, Japan). The ratio of emitted fluorescence signals from the Fura-2/AM loaded cardiomyocytes at 510 nm was recorded. The Ca^2+^ transient parameters including the [Ca^2+^]i transient amplitude, rising rate, and decay rate, as well as the diastolic Ca^2+^ level were recorded during electrical pacing (1 hertz field-stimulation, 10 millisecond duration and 15 volts). The ratio of the emission wavelengths is directly related to the amount of cardiac [Ca^2+^]i^74^. The fluorescence ratio data was processed and stored in a computer using Xcellence imaging software (Olympus, Tokyo, Japan)^71^,^72^.

### Western blot analysis

The heart tissues were extracted in a homogenizer in a lysis buffer (20 mM Tris–HCl, 1 mM Na_3_VO_4_, 5 mM NaF, and 1% proteinase inhibitor) at 4 °C, and subsequently centrifuged at 13,500 rpm for 10 minutes. The total protein of 40 mg was mixed with the loading buffer (1 mg/mL), boiled for 10 minutes and loaded onto 10% gradient sodium dodecyl sulfate (SDS)-polyacrylamide gels. Proteins were transferred to a nitrocellulose blotting membrane (GE Healthcare Life Sciences, Freiburg, Germany). Immunoblots were blocked with either 5% of bovine serum albumin or 5% of skimmed milk in Tris-buffered saline (pH 7.4) containing 0.1% Tween 20 (TBST) for 1 hour at room temperature. Then, immunoblots were exposed to the primary antibodies including the iron transporter protein DMT1 (ab55812; Abcam, Cambridge, MA, USA) and Zip14 (ab106568; Abcam, Cambridge, MA, USA), iron regulatory protein ferroportin (ab85370; Abcam, Cambridge, MA, USA) and hepcidin (ab75883; Abcam, Cambridge, MA, USA), Ca^2+^ cycling protein LTCC (C1603; Sigma-Aldrich Co. LLC., Saint Louis, MO, USA.), TTCC (sc25691; Santa Cruz Biotechnology Inc., Dallas, Texas, USA), SERCA (sc30110; Santa Cruz Biotechnology Inc., Dallas, Texas, USA) and NCX (sc32881; Santa Cruz Biotechnology Inc., Dallas, Texas, USA), and β-actin (sc47778; Santa Cruz Biotechnology Inc., Dallas, Texas, USA) for a loading control at 4 °C overnight. After that, the membrane was washed five times with TBST and then incubated with the horseradish peroxidase-conjugated secondary antibody at room temperature for 1 hour. After washing the membrane again five times with TBST, the blots were visualized with an enhanced chemiluminescence (ECL) reagent and exposed by using ChemiDoc^TM^ Touch Imaging System (Life science AP, Bio-Rad, CA, USA). The images of the western blots were analysed using the ImageJ (NIHimage) analysis software^17^,^75^.

### Statistical analysis

Data were expressed as mean ± standard error of mean (SEM). The data were processed using SPSS (Statistical Package for Social Sciences, Chicago, IL, USA) release 22.0 for Windows. Differences between groups were tested for via One-way ANOVA analyses. Significant difference between groups was assumed if *P* values < 0.05.

## Additional Information

**How to cite this article**: Wongjaikam, S. *et al*. Restoring the impaired cardiac calcium homeostasis and cardiac function in iron overload rats by the combined deferiprone and N-acetyl cysteine. *Sci. Rep.* **7**, 44460; doi: 10.1038/srep44460 (2017).

**Publisher's note:** Springer Nature remains neutral with regard to jurisdictional claims in published maps and institutional affiliations.

## Supplementary Material

Supplementary Information

## Figures and Tables

**Table 1:** Summary the effects of all pharmacological interventions in each cardiac parameter.

Cardiac parameters	ND	HFeV	HFeDFO	HFeDFP	HFeDFX	HFeNAC	HFeDFP + NAC
**Echocardiography**							
- % LVEF	+++	+	++	++	++	++	+++
**Intracellular Ca**^**2+**^ **transients**							
- Amplitude	+++	+	++	++	++	++	+++
- Rising rate	+++	+	++	++	++	++	+++
- Decay rate	+++	+	+++	+++	+++	+++	+++
- Diastolic Ca^2+^ level	+++	++++	+++	+++	+++	+++	+++
**The levels of Ca**^**2+**^ **cycling protein**							
- LTCC	+++	+++	+++	+++	+++	+++	+++
- TTCC	+	++	++	++	++	++	++
- SERCA	+++	+	+++	+++	+++	+++	+++
- NCX	+++	+++	+++	+++	+++	+++	+++
**The levels of iron transporter and regulatory proteins**							
- DMT1	+++	+++	+++	+++	+++	+++	+++
- ZIP14	+++	+++	+++	+++	+++	+++	+++
- Hepcidin	+++	+++	+++	+++	+++	+++	+++
- Ferroportin	+++	+++	+++	+++	+++	+++	+++
