# Supplementary material for: Restoring the impaired cardiac calcium homeostasis and cardiac function in iron overload rats by the combined deferiprone and N-acetyl cysteine
Source: Sci Rep. 2017 Mar 13;7:44460. doi: 10.1038/srep44460 (PMC5347387; doi:10.1038/srep44460)
Supplement: Supplementary Information [file srep44460-s1.pdf]

Supplementary Information file

Title: Restoring the impaired cardiac calcium homeostasis and cardiac function in iron overload rats by the combined deferiprone and N-acetyl cysteine

Authors: Suwakon Wongjaikam, Sirinart Kumfu, Juthamas Khamseekaew, Siriporn C. Chattipakorn, and Nipon Chattipakorn*

Supplementary Methods

Determination of non-transferrin bound iron (NTBI) level. The levels of plasma NTBI was measured using nitrilotriacetic acid disodium salt (NTA) chelation/flow cytometry [1,2] with some modifications for determining free iron in plasma of all rats in each group. Plasma NTBI was performed according to the method described previously [3].

Determination of malondialdehyde (MDA) level. The levels of plasma and cardiac MDA were measured using the high-performance liquid chromatography (HPLC) method [4] for assessing systemic and cardiac oxidative stress in iron overload rats in each group. Plasma and cardiac MDA concentration were performed according to the method described previously [3].

Determination of cardiac iron concentration. Cardiac iron concentration was measured using a colorimetric method [5,6] for determining non-heme iron in heart tissues of rats in each group. Cardiac iron concentration was performed according to the method described previously [3].

References

1. Singh, S., Hider, R.C. & Porter, J.B. A direct method for quantification of non-transferrin-bound iron. *Analytical biochemistry* **186**, 320-323 (1990).
2. Ma, Y., *et al.* A novel method for non-transferrin-bound iron quantification by chelatable fluorescent beads based on flow cytometry. *The Biochemical journal* **463**, 351-362 (2014).
3. Wongjaikam, S., *et al.* Combined Iron Chelator and Antioxidant Exerted Greater Efficacy on Cardioprotection Than Monotherapy in Iron-Overloaded Rats. *PloS one* **11**, e0159414 (2016).
4. Grotto, D., *et al.* Rapid quantification of malondialdehyde in plasma by high performance liquid chromatography-visible detection. *Journal of pharmaceutical and biomedical analysis* **43**, 619-624 (2007).
5. Rebouche, C.J., Wilcox, C.L. & Widness, J.A. Microanalysis of non-heme iron in animal tissues. *Journal of biochemical and biophysical methods* **58**, 239-251 (2004).
6. Kumfu, S., Chattipakorn, S., Chinda, K., Fucharoen, S. & Chattipakorn, N. T-type calcium channel blockade improves survival and cardiovascular function in thalassemic mice. *European journal of haematology* **88**, 535-548 (2012).

Supplementary Table 1. Effects of all pharmacological interventions on iron status in iron-overloaded rats.

Iron status	ND	HFeV	HFeDFO	HFeDFP	HFeDFX	HFeNAC	HFeDFP+NAC
Plasma NTBI level (μM)	0.00±0.00	0.61±0.12*	0.28±0.07*,†	0.36±0.06*,†	0.35±0.05*,†	0.38±0.06*,†	0.31±0.05*,†
Plasma MDA level (μM)	1.85±0.23	14.84±1.91*	8.47±1.28*,†	9.08±1.00*,†	10.23±0.61*,†	8.86±1.00*,†	8.72±1.14*,†
Cardiac iron concentration (μM/mg protein)	8.04±0.47	48.00±7.91*	23.68±1.34*,†,‡	23.58±1.24*,†,‡	24.43±1.16*,†,‡	25.69±2.08*,†,‡	14.23±2.10†
Cardiac MDA concentration (μM/mg protein)	3.39±0.96	39.20±11.97*	20.55±3.34*,†,‡	20.31±5.44*,†,‡	20.62±6.15*,†,‡	5.11±1.08†	3.85±0.76†

MDA=malondialdehyde; *P < 0.05 vs. ND control, †P < 0.05 vs. HFeV, ‡P < 0.05 vs. HFeDFP+NAC.

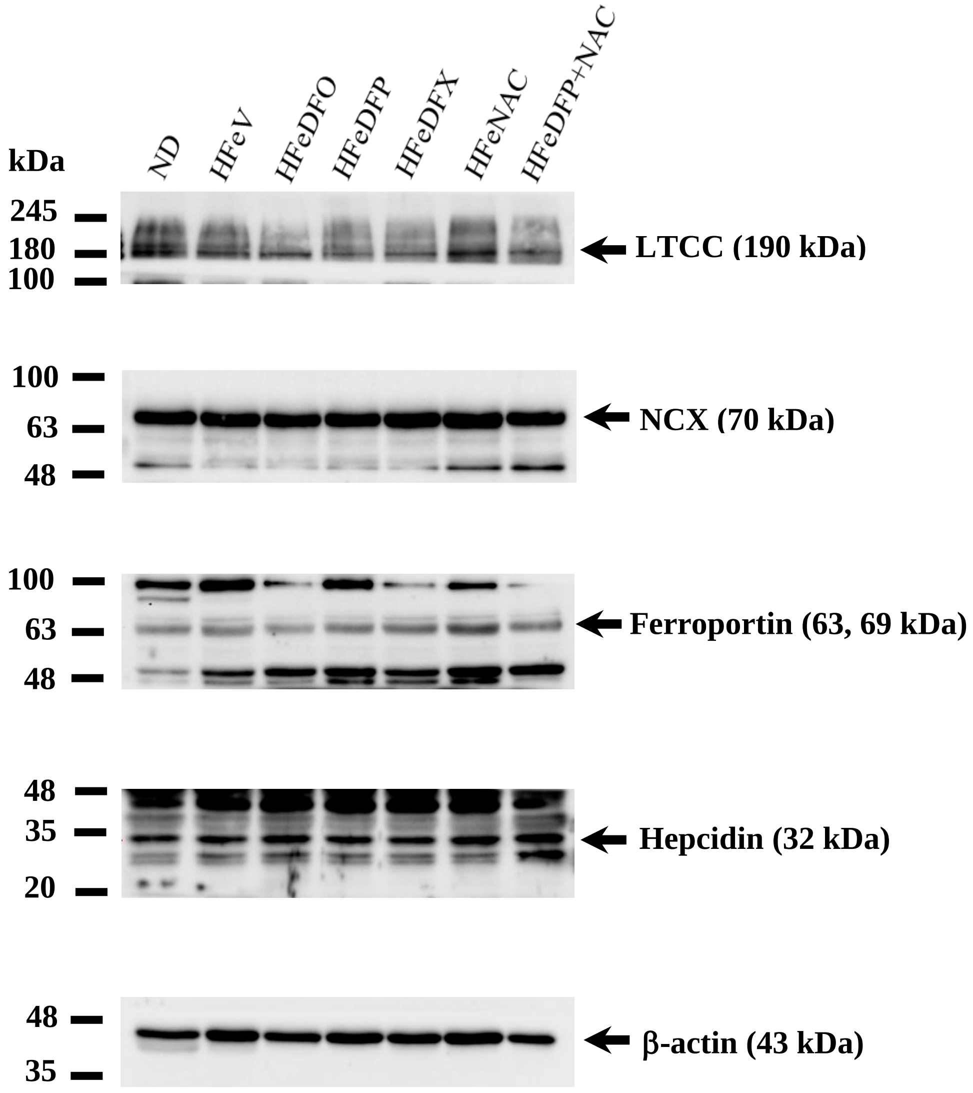

Supplementary Figure 1. Full-length blots of data shown in Fig. 3a,d and 4c,d.

The gels were initially cut ranged from 20 kDa to 245 kDa.

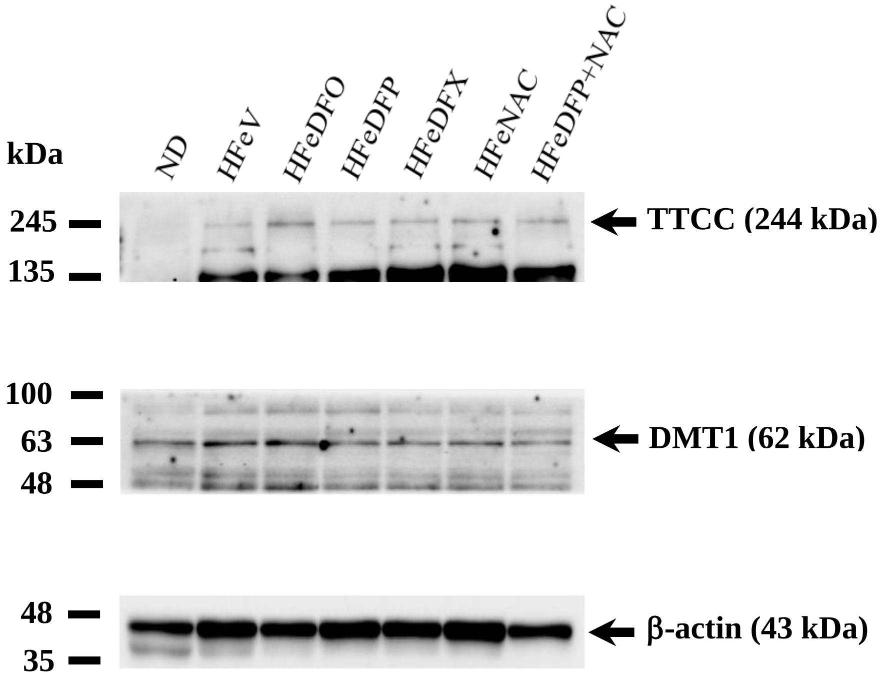

Supplementary Figure 2. Full-length blots of data shown in Fig. 3b and 4a.

The gels were initially cut ranged from 35 kDa to 245 kDa.

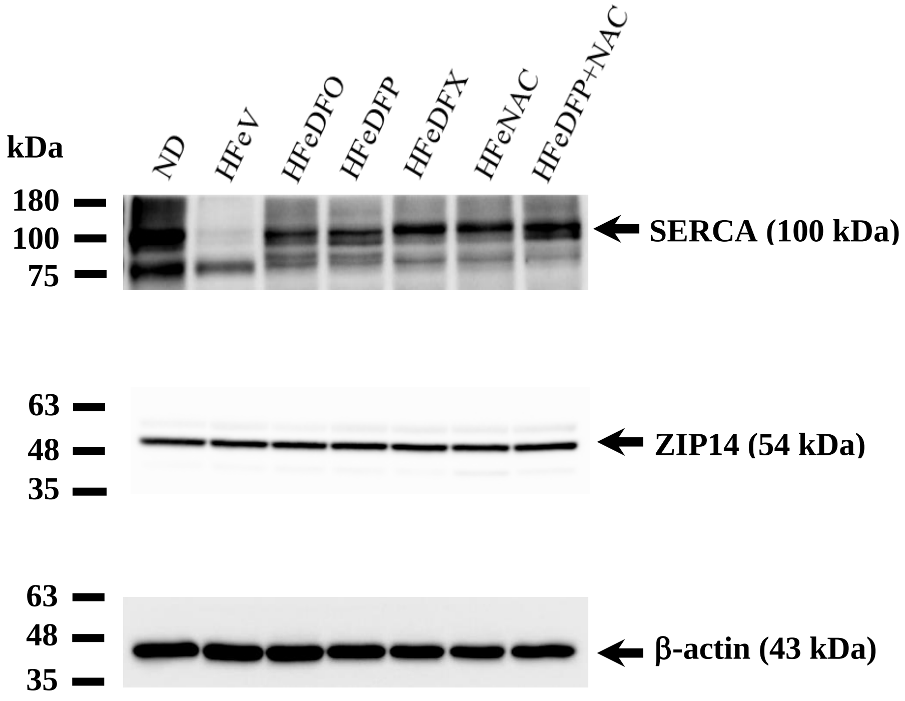

Supplementary Figure 3. Full-length blots of data shown in Fig. 3c and 4b.

The gels were initially cut ranged from 35 kDa to 180 kDa.
